# Exploring vibrational properties, Rashba spin splitting, and phonon-limited carrier mobility in Janus WBPX_2_ (X = S, Se, Te) monolayers

**DOI:** 10.1039/d6ra02529c

**Published:** 2026-05-20

**Authors:** Tuan V. Vu, Le T. Hoa, Huynh V. Phuc, A. I. Kartamyshev, Nguyen N. Hieu, Vo T. T. Vi

**Affiliations:** a Laboratory for Computational Physics, Institute for Computational Science and Artificial Intelligence, Van Lang University Ho Chi Minh City Vietnam tuan.vu@vlu.edu.vn; b Faculty of Mechanical, Electrical, and Computer Engineering, Van Lang School of Technology, Van Lang University Ho Chi Minh City Vietnam; c Faculty of Physics and Chemistry, The University of Danang – University of Science and Education Da Nang 550000 Vietnam; d Division of Physics, School of Education, Dong Thap University Dong Thap 870000 Vietnam; e Institute of Research and Development, Duy Tan University Da Nang 550000 Vietnam hieunn@duytan.edu.vn; f Faculty of Natural Science, Duy Tan University Da Nang 550000 Vietnam; g Faculty of Basic Sciences, University of Medicine and Pharmacy, Hue University Hue Vietnam vothituyetvi@hueuni.edu.vn

## Abstract

The search for multifunctional two-dimensional Janus semiconductors has attracted increasing attention due to their potential in next-generation nanoelectronic and spintronic technologies. In this work, we present a comprehensive first-principles investigation of the structural stability, vibrational characteristics, electronic properties, and carrier transport behavior of Janus WBPX_2_ (X = S, Se, Te) monolayers. The calculated cohesive energies, phonon dispersions, and *ab initio* molecular dynamics simulations confirm the energetic and thermodynamic stability of all proposed structures. Electronic structure analysis reveals that WBPX_2_ monolayers are semiconductors with moderate band gaps. When spin–orbit coupling is included, noticeable spin splitting appears in the electronic bands, and the WBPX_2_ monolayers exhibit a pronounced Rashba effect, indicating strong potential for spintronic applications. Carrier transport analysis further shows that the intrinsic carrier mobility in these systems is relatively low and is primarily limited by acoustic deformation potential scattering. These findings provide deeper insight into the fundamental transport behavior of Janus WBPX_2_ monolayers and highlight their promising suitability for emerging two-dimensional electronic and spintronic technologies.

## Abstract 2

The search for multifunctional two-dimensional Janus semiconductors has attracted increasing attention due to their potential in next-generation nanoelectronic and spintronic technologies.

## Graphical abstract

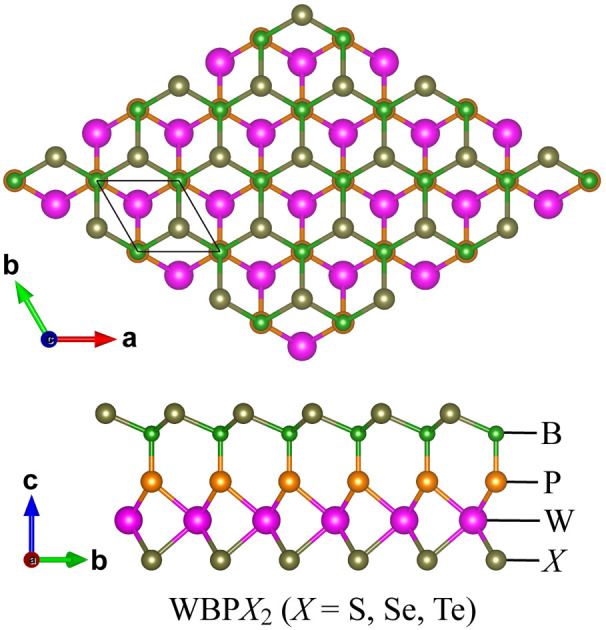

## 1 Introduction

Two-dimensional (2D) materials have attracted sustained interest in condensed matter physics and materials science due to their reduced dimensionality, strong quantum confinement, and highly tunable physical properties.^1,2^ Beyond graphene,^3^ a wide variety of layered 2D systems have been explored, revealing diverse electronic, optical, mechanical, and transport behaviors that are absent in their bulk counterparts.^4–7^ In recent years, particular attention has been devoted to 2D Janus materials, which are characterized by intrinsic out-of-plane asymmetry arising from the substitution of different atomic species on the two sides of a monolayer.^8,9^ This broken mirror symmetry gives rise to built-in electric fields, enabling functionalities that cannot be realized in the vertically symmetric counterparts, such as spin splitting induced by spin–orbit coupling, efficient charge separation, and enhanced out-of-plane piezoelectric responses.^10–13^

From the perspective of device applications, carrier mobility is widely regarded as a key parameter governing the performance of 2D materials in nanoelectronic and optoelectronic devices. It directly determines charge transport efficiency, switching speed, and power dissipation, thereby setting intrinsic limits on device operation. In low-dimensional systems, transport properties are particularly sensitive to lattice vibrations, reduced screening, and anisotropic bonding, which often result in transport behavior markedly different from that of conventional bulk semiconductors. Consequently, understanding the intrinsic limits of carrier mobility in 2D materials is essential for evaluating their technological potential.

Carrier transport is strongly influenced by various scattering mechanisms, among which phonon scattering plays a dominant role under typical operating conditions. In 2D materials, the effect of phonon scattering is often amplified due to reduced dimensionality and enhanced electron–phonon interactions. Traditional approaches for estimating carrier mobility, such as deformation potential (DP) theory,^14^ are widely used because of their conceptual simplicity and low computational cost. However, these simplified models typically consider only long-wavelength acoustic phonon scattering and neglect other relevant channels, including piezoelectric interactions, polar optical phonon scattering, and impurity-related processes, which can be significant in polar or structurally asymmetric systems.^15,16^ To overcome these limitations, more rigorous theoretical frameworks have been developed. The semiclassical Boltzmann transport equation (BTE), when combined with first-principles calculations, provides a quantitative description of carrier transport by explicitly incorporating electron–phonon interactions.^17^ Within this approach, density functional perturbation theory (DFPT) enables the direct calculation of phonon spectra and electron–phonon coupling matrix elements from first principles,^18^ forming the basis for predictive mobility calculations. Recent advances employing Wannier function interpolation techniques, as implemented in codes such as EPW, have further enabled highly accurate evaluations of carrier lifetimes and mobilities on dense Brillouin zone grids.^19^ Together, these theoretical and computational approaches constitute a comprehensive toolkit for investigating carrier transport in 2D materials, enabling more reliable predictions of intrinsic mobility and facilitating the rational design of next-generation electronic and spintronic devices.

Janus monolayers represent an especially attractive platform for such investigations. Their intrinsic structural asymmetry not only modifies the electronic band structure, often in conjunction with strong spin–orbit coupling effects, but also introduces additional scattering pathways associated with polarity and piezoelectricity.^20^ Recent studies on various Janus material families have consistently shown that carrier mobility is frequently limited by phonon-related processes, with acoustic phonon scattering emerging as the dominant mechanism in many cases, while other channels may become significant depending on carrier type, concentration, and chemical composition.^21–23^ These findings highlight the necessity of treating carrier transport in Janus systems within a unified framework that captures the interplay among lattice dynamics, electronic structure, and symmetry breaking. In this work, we introduce a family of Janus WBPX_2_ monolayers (X = S, Se, Te) and investigate their fundamental physical properties using first-principles calculations. Although the broken out-of-plane mirror symmetry in Janus structures generally generates an intrinsic electric field, the particular interest in the WBPX_2_ family arises from the distinctive properties expected from its chemical composition. In particular, the incorporation of the heavy transition metal tungsten (W) is anticipated to produce strong spin–orbit coupling, which can lead to significant spin splitting and thereby enhance the potential of these materials for spintronic applications. Particular attention is devoted to the intrinsic charge transport characteristics, where carrier mobility is evaluated by explicitly accounting for multiple phonon-related scattering mechanisms within a realistic transport framework. In addition to transport behavior, the structural stability of the WBPX_2_ systems is assessed through cohesive energy analysis, phonon dispersion calculations, and *ab initio* molecular dynamics simulations. The electronic structures are analyzed using both semilocal and hybrid exchange–correlation functionals, with spin–orbit coupling effects included to capture relativistic contributions relevant to Janus asymmetry. Vibrational properties and Raman-active modes are further explored to provide insight into lattice dynamics and experimental identification. Overall, the present results clarify the interplay among structural asymmetry, lattice vibrations, and charge carrier scattering in Janus WBPX_2_ monolayers, and offer theoretical guidance for their potential applications in next-generation electronic and polarization-sensitive devices.

## 2 Computational method

First-principles calculations in this study were carried out using density functional theory (DFT) within the Vienna it *ab initio* simulation package (VASP) framework.^24,25^ The interaction between electrons and ions was treated using the projector augmented-wave (PAW) method.^26,27^ We performed structural relaxations using a generalized gradient approximation (GGA) with the Perdew–Burke–Ernzerhof (PBE) functional.^28^ Convergence was reached when residual atomic forces fell below 10^−3^ eV Å^−1^ and the total energy stabilized within 10^−6^ eV. To obtain more accurate electronic band structures and band gap values, additional simulations were carried out using the Heyd–Scuseria–Ernzerhof (HSE06) functional.^29^ Relativistic effects were incorporated by including spin–orbit coupling (SOC) self-consistently.^30^ A plane-wave cutoff energy of 650 eV and a (12 × 12 × 1) Monkhorst–Pack *k*-point grid were employed. Rigorous convergence tests confirm that the employed *k*-point mesh yields negligible differences in the obtained results compared to a dense, odd-numbered *Γ*-centered grid. Spurious periodic interactions were eliminated using a 30 Å vacuum space, while dipole corrections accounted for the inherent asymmetry of the structure. Phonon dispersion relations were calculated using the finite-displacement method as implemented in PHONOPY,^31^ employing a 5 × 5 × 1 supercell expansion with an atomic displacement amplitude of 0.01 Å. *Ab initio* molecular dynamics (AIMD) simulations were conducted at 300 K within the canonical (*NVT*) ensemble using a Nosé thermostat to evaluate thermal stability. Carrier transport properties were investigated using the AMSET code,^32^ which includes scattering from acoustic deformation potentials, ionized impurities, piezoelectric interactions, and polar optical phonons. The total carrier mobility was obtained using Matthiessen's rule.^23^ Raman spectra were calculated based on the macroscopic dielectric tensor.^33^

## 3 Results and discussion

### 3.1 Structure, stability, and Raman spectra

**Fig. 1:**
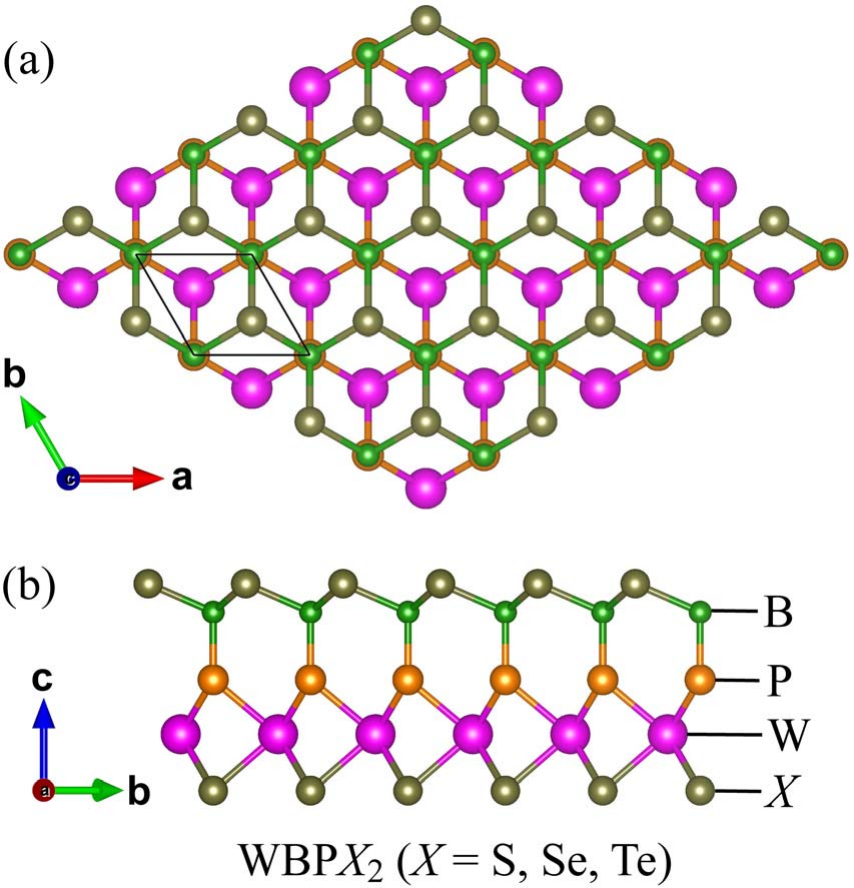
Optimized crystal structures of Janus WBPX_2_ (X = S, Se, and Te) monolayers in the top (a) and side (b) views.

The optimized geometries of the Janus WBPX_2_ (X = S, Se, Te) monolayers are depicted in Fig. 1. These WBPX_2_ systems constitute a class of 2D materials with a hexagonal crystal lattice and exhibit a pronounced structural asymmetry along the out-of-plane direction. The lack of inversion symmetry results in WBPX_2_ monolayers crystallizing in the *P*3*m*1 space group. Based on first-principles structural relaxation, the optimized lattice parameters of the Janus WBPX_2_ systems are listed in Table 1. The calculated in-plane lattice constants are 3.16, 3.29, and 3.50 Å for the WBPS_2_, WBPSe_2_, and WBPTe_2_ configurations, respectively. In addition, the monolayer thickness, defined as the vertical distance between the outermost atomic layers, is determined to be 5.84, 5.96, and 6.15 Å for the WBPS_2_, WBPSe_2_, and WBPTe_2_ systems, respectively. The gradual increase in both the lattice constant and the thickness reflects the influence of atomic size on the structural characteristics of the WBPX_2_ monolayers, indicating that their geometric parameters can be effectively modulated through compositional variation.

**Table 1:** Lattice constant *a* (Å), bond length *d* (Å), monolayer thickness Δ*h* (Å) and cohesive energy *E*_coh_ (eV per atom) of Janus WBPX_2_ monolayers

	*a*	*d*_B–X_	*d*_B–P_	*d*_P–W_	*d*_W–X_	Δ*h*	*E*_coh_
WBPS_2_	3.16	2.00	1.91	2.39	2.42	5.84	−6.89
WBPSe_2_	3.29	2.10	1.88	2.40	2.55	5.96	−6.51
WBPTe_2_	3.50	2.26	1.91	2.44	2.74	6.15	−6.12

To examine the structural stability of the proposed Janus WBPX_2_ monolayers, we analyze their cohesive energy, which reflects the overall strength of interatomic interactions within the crystal lattice. Cohesive energy is widely regarded as a fundamental thermodynamic descriptor, as it quantifies the energy gain associated with the formation of a solid from its isolated constituent atoms. We calculate the value of the cohesive energy using the following expression:1
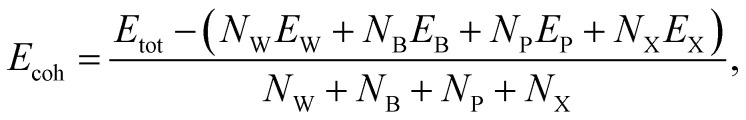
where *E*_tot_ refers to the total energy of the Janus WBPX_2_ monolayer. *E*_W_, *E*_B_, *E*_P_, and *E*_X_ are the energies of the isolated W, B, P, and X, respectively. *N*_W_, *N*_B_, *N*_P_, and *E*_X_ indicate the numbers of W, B, P, and X in the unit cell, respectively.

The calculated cohesive energies for the WBPS_2_, WBPSe_2_, and WBPTe_2_ materials are −6.89, −6.51, and −6.12 eV per atom, respectively. The consistently negative formation energies confirm the energetic stability of these monolayers, with their large magnitudes reflecting robust chemical bonding among the constituent W, B, P, and X atoms. Furthermore, the gradual reduction in the absolute cohesive energy can be correlated with variations in atomic size and bonding characteristics of the chalcogen component, which influence the overall lattice cohesion. Notably, the obtained cohesive energies fall within the typical range reported for experimentally realized 2D materials. For comparison, graphene possesses a cohesive energy of about −7.6 eV per atom,^34^ while Janus transition metal dichalcogenides, such as WTeSe, exhibit a cohesive energy of −5.06 eV per atom,^35^ implying that the WBPX_2_ monolayers possess sufficient energy stability and may represent viable candidates for further theoretical exploration and potential experimental realization.

**Fig. 2:**
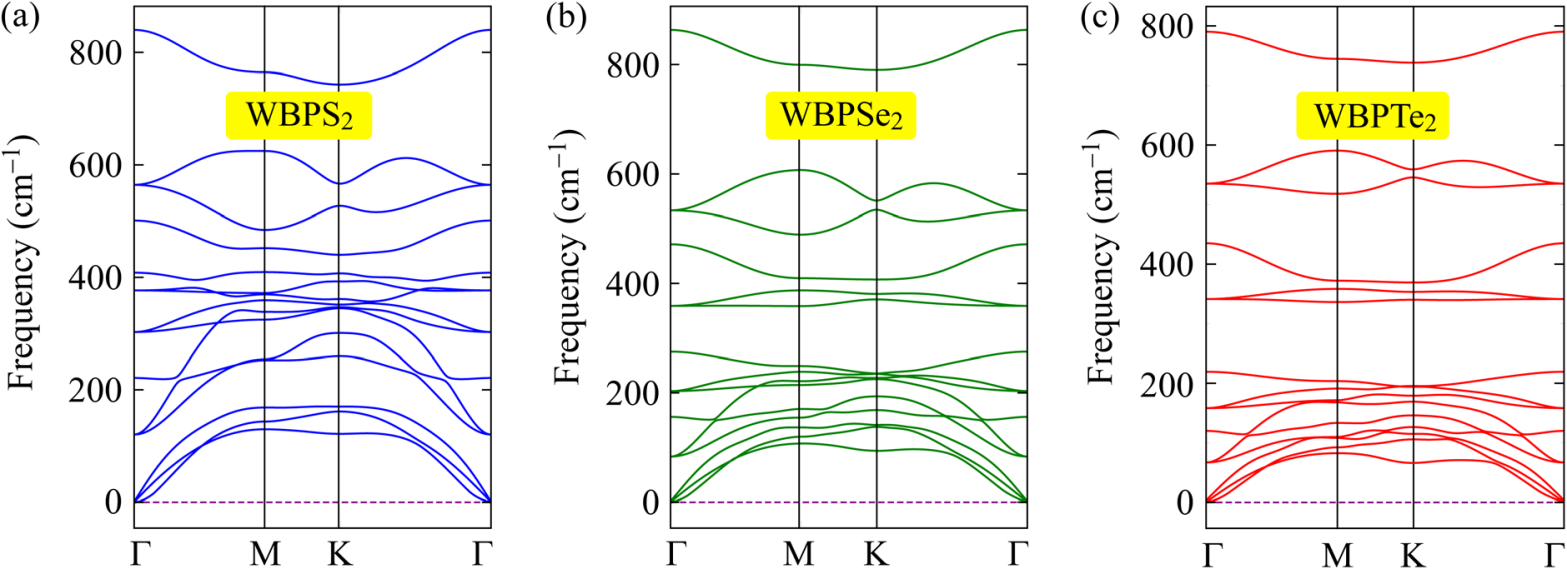
Phonon spectra of WBPS_2_ (a), WBPSe_2_ (b), and WBPTe_2_ (c) monolayers.

**Fig. 3:**
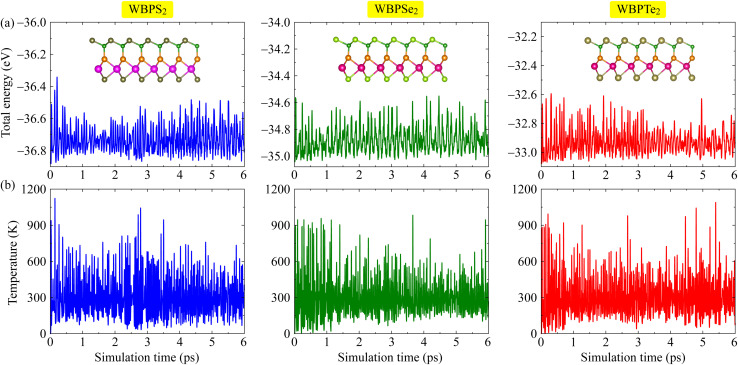
AIMD simulations of Janus WBPX_2_ monolayers at room temperature: time-evolution of (a) the total energy and (b) the temperature. The insets in (a) display snapshots of the atomic configurations at the end of the simulation.

To further examine the structural robustness of the proposed Janus monolayers, we investigated their dynamical and thermal stabilities through phonon dispersion calculations and *ab initio* molecular dynamics (AIMD) simulations. Phonon spectra were calculated to probe lattice vibrational behavior and to confirm whether the optimized geometries correspond to stable configurations. As each primitive unit cell consists of five atoms, the resulting phonon dispersions comprise fifteen vibrational branches, including three acoustic modes in the low-frequency region and twelve optical modes at higher frequencies, as presented in Fig. 2. Across the entire Brillouin zone, no imaginary phonon modes are observed, indicating that the studied structures are dynamically stable and free from lattice instabilities. Notably, the acoustic and optical branches partially overlap in frequency, suggesting pronounced acoustic–optical phonon interactions, which may play an important role in phonon scattering processes and thermal transport characteristics. In addition, a distinct phonon gap appears within the optical frequency range, which can be attributed to the mass asymmetry inherent to the Janus configuration and may offer potential for phononic management applications. AIMD simulations were performed to evaluate the finite-temperature stability of the proposed Janus monolayers at room temperature. The time evolution of the system temperature during the 6 ps is illustrated in Fig. 3. Throughout the simulation, the temperature remains well regulated around 300 K, exhibiting only moderate and smooth fluctuations that are characteristic of thermally equilibrated systems. Importantly, the final atomic configurations show no signs of structural degradation, including bond rupture, atomic rearrangement, or lattice distortion. The preservation of the original crystal framework under thermal agitation confirms that the studied Janus monolayers maintain robust thermal and structural stability under ambient conditions.

Raman spectroscopy is widely employed as a nondestructive technique to investigate the vibrational properties of low-dimensional materials, as the position and intensity of Raman-active modes are strongly influenced by lattice symmetry and atomic configurations. Since no experimental Raman spectra for the studied materials are currently available in the literature, the spectra presented here are derived entirely from first-principles DFT calculations and serve as theoretical predictions to guide future experiments. The Raman-active vibrational modes are dictated by the variation of the polarizability tensor with respect to the normal vibrational coordinates, in accordance with Raman selection rules. In the studied Janus structures, the breaking of out-of-plane mirror symmetry along the *z*-axis induces a built-in dipole moment, which profoundly alters the macroscopic polarizability of the lattice. Consequently, the derivative of the polarizability tensor with respect to the normal vibrational coordinates is modified, relaxing the strict Raman selection rules typical of the parent materials. Therefore, these calculated Raman spectra not only provide characteristic fingerprints for material identification but also offer fundamental insight into the vibrational and bonding properties of the investigated systems. In this part, by analyzing the Raman-active modes and their corresponding intensities, we establish a theoretical reference that supports the interpretation of experimental measurements and provides deeper insight into the dynamical properties of the material. The crystal structure of the Janus WBPX_2_ monolayers belongs to the *P*3*m*1 symmetry group, as discussed earlier. The lattice symmetry determines the characteristics of the vibrational modes at the *Γ* point of the Brillouin zone. The optical phonon spectrum at this point can be decomposed into four nondegenerate A_1_ modes and four doubly degenerate E modes, which can be expressed by the following irreducible representation:

2 *Γ*_optical_ = 4A_1_ + 4E.

**Fig. 4:**
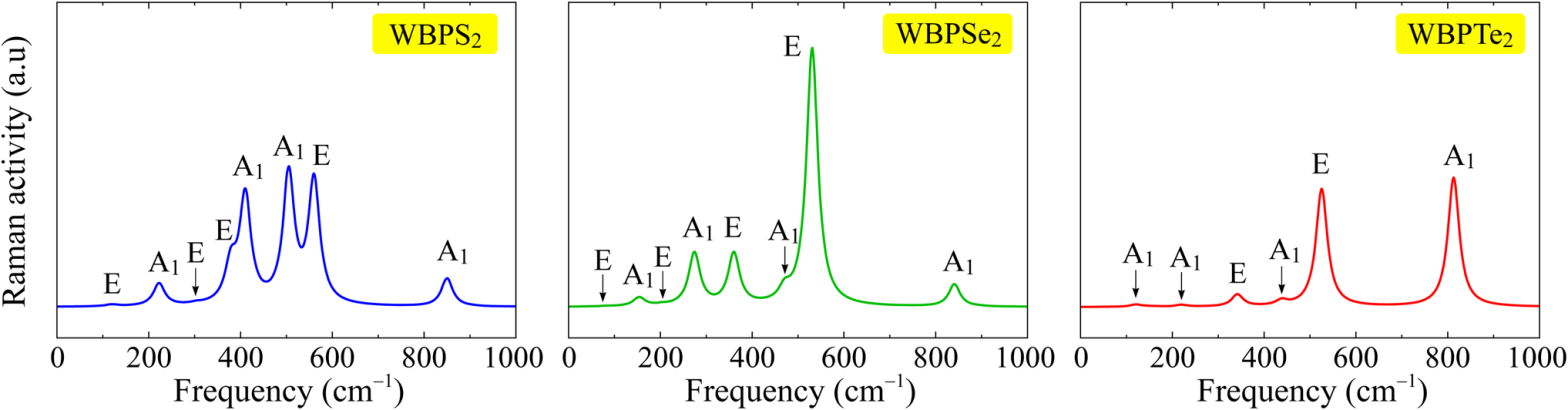
Calculated Raman spectra of Janus WBPS_2_, WBPSe_2_, and WBPTe_2_ monolayers.

According to the Raman selection rules for the *P*3*m*1 space group, both A_1_ and E modes are Raman-active. Consequently, all optical phonon modes predicted from symmetry considerations are, in principle, detectable through Raman scattering measurements. The simulated *Γ*-point Raman spectra for the Janus WBPX_2_ monolayers are displayed in Fig. 4. Based on the phonon mode decomposition given in eqn (2), the system is expected to exhibit eight Raman-active vibrational modes. However, the calculated spectra reveal that several of these modes possess extremely weak Raman intensities, which make them difficult to resolve or practically invisible in the simulated Raman spectra. The calculated Raman spectra reveal notable differences in the relative intensities of the vibrational modes among the three monolayers. In WBPS_2_ monolayer, prominent A_1_ and E Raman modes are observed between 400 and 600 cm^−1^. Conversely, a sharp peak corresponding to the E mode is observed at 530 cm^−1^, showing a significantly higher intensity than other active modes in the Janus WBPSe_2_ monolayer. Janus WBPTe_2_ presents a different profile that while most Raman-active modes remain relatively weak, two prominent peaks corresponding to the E and A modes at 525 and 810 cm^−1^, respectively stand out with markedly superior intensities.

### 3.2 Electronic characteristics

**Fig. 5:**
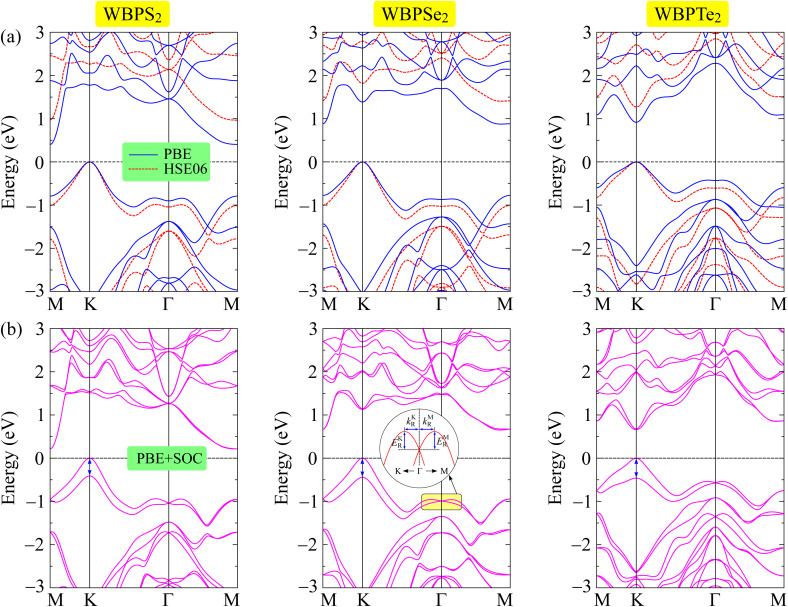
Band diagrams of Janus WBPX_2_ monolayers calculated using (a) the PBE/HSE06 and (b) PBE + SOC methods. The insets in (b) provide a schematic of the Rashba effect, defining the Rashba energy (*E*_R_) and the momentum offset (*k*_R_).

The electronic characteristics of the three investigated WBPX_2_ monolayers were analyzed through their calculated band dispersions along the high-symmetry directions in the first Brillouin zone. The electronic band structures of the Janus WBPX_2_ monolayers calculated at different theoretical levels are illustrated in Fig. 5. The obtained results confirm that all three structures possess finite band gaps, indicating semiconducting behavior. Within the PBE framework, the WBPS_2_ and WBPSe_2_ compounds exhibit indirect band gaps as shown in Fig. 5(a). Both systems exhibit an indirect-gap nature, with the valence band maximum (VBM) and conduction band minimum (CBM) situated at the *K* and *M* points of the first Brillouin zone, respectively. By contrast, the WBPTe_2_ monolayer displays a direct band gap at the *K* point. The transition from an indirect band gap in WBPS_2_ and WBPSe_2_ to a direct band gap in WBPTe_2_ is driven by a synergistic combination of structural expansion and modified orbital hybridization. As summarized in Table 2, the band gaps calculated at the PBE level are 0.40, 0.87, and 0.92 eV for WBPS_2_, WBPSe_2_, and WBPTe_2_, respectively.

**Table 2:** Calculated PBE, HSE06, and PBE + SOC band gaps *E*_g_, Zeeman spin-splitting energy at the valence band *λ*_v_, and Rashba parameters (*E*_R_, *k*_R_, and *α*_R_) of Janus WBPX_2_ monolayers

	*E* ^PBE^_g_ (eV)	*E* ^HSE06^_g_ (eV)	*E* ^PBE+SOC^_g_ (eV)	*λ*_v_ (eV)	*E* ^R^_*K*_ (meV)	*E* ^R^_*M*_ (meV)	*k* ^R^_*K*_ (Å^−1^)	*k* ^R^_*M*_ (Å^−1^)	*α* ^R^_*K*_ (meV Å)	*α* ^R^_*M*_ (meV Å)
WBPS_2_	0.40	0.96	0.22	0.41	100.10	92.70	0.227	0.197	881.94	941.12
WBPSe_2_	0.87	1.40	0.65	0.44	32.20	30.20	0.167	0.156	385.63	387.18 3
WBPTe_2_	0.92	1.26	0.66	0.46	31.50	29.60	0.121	0.105	520.66	563.81

To improve the description of the electronic structure, we employed the hybrid HSE06 functional. While the band dispersion remains qualitatively similar to the PBE results, the band gaps undergo a pronounced widening, correcting the characteristic underestimation inherent in semilocal exchange–correlation functionals. The HSE06 functional yields increased band gaps of 0.96, 1.40, and 1.26 eV for WBPS_2_, WBPSe_2_, and WBPTe_2_, respectively, owing to the superior treatment of electronic exchange within the hybrid framework. Notably, the indirect band gaps of WBPS_2_ and WBPSe_2_, along with the direct-gap nature of WBPTe_2_, are maintained under the HSE06 functional. This suggests that while quantitative gap values are significantly corrected, the qualitative electronic landscape remains robust against the specific choice of exchange–correlation functional.

An accurate description of the electronic structure of 2D materials, particularly those incorporating heavy elements, requires the explicit consideration of spin–orbit coupling (SOC). Owing to relativistic effects that become significant in such systems, SOC can substantially modify the band dispersion and lift spin degeneracy, leading to characteristic spin splitting near the high-symmetry points. These SOC-induced features govern charge transport and spin polarization, and possible spin–valley coupling phenomena, thereby directly affecting the suitability of these materials for spintronic and related applications. Considering the substantially increased computational cost of hybrid functional calculations with SOC, the SOC effects were evaluated at the PBE level, which is sufficient to capture the essential spin–orbit-induced modifications in the electronic structure, while HSE06 calculations were mainly used to obtain improved band-gap estimations. As illustrated in Fig. 5(b), the incorporation of spin–orbit coupling (PBE + SOC) leads to a discernible reduction in the band gaps compared to standard PBE results. Specifically, the PBE + SOC band gaps of WBPS_2_, WBPSe_2_, and WBPTe_2_ are found to be 0.22, 0.65, and 0.66 eV, respectively, confirming the significant influence of SOC on the electronic states. In addition, the inclusion of SOC removes spin degeneracy and induces pronounced Zeeman-type spin splitting at the valence band maximum. The calculated Zeeman spin-splitting energies (*λ*_v_) are 0.41, 0.44, and 0.46 eV for WBPS_2_, WBPSe_2_, and WBPTe_2_, respectively, indicating strong spin–orbit interactions in all three systems. Moreover, clear Rashba-type spin splitting is observed around the high-symmetry region of the Brillouin zone. The strength of the Rashba effect is evaluated through the Rashba parameters *α*_R_^*M*^ and *α*_R_^*K*^ along the *Γ*–*M* and *Γ*–*K* directions, derived from the momentum offset *k*_R_ and Rashba energy *E*_R_ extracted from the spin-resolved band structures using the relation *α*_R_ = 2*E*_R_/*k*_R_.^36,37^ For the S_2_ monolayer, remarkably large Rashba parameters, *α*_R_^*M*^ = 881.94 and *α*_R_^*K*^ = 941.12 meV Å, are obtained along the *Γ*–*M* and *Γ*–*K* directions, respectively. These values demonstrate a robust SOC-induced spin splitting arising from the broken inversion symmetry. For Janus WBPSe_2_, the Rashba parameters (*α*_R_^*M*^ = 385.63 and *α*_R_^*K*^ = 387.18 meV Å) are remarkably similar, indicating that the SOC-induced splitting is essentially isotropic across the *Γ*–*M* and *Γ*–*K* directions. Meanwhile, WBPTe_2_ shows substantial Rashba parameters of 520.66 and 563.81 meV Å along *Γ*–*K* and *Γ*–*M*, respectively. Interestingly, the calculated Rashba parameters of WBPX_2_ monolayers are substantially higher than the values reported for several previously studied 2D systems, including SnSeTe (273 meV Å)^38^ and WSiGeN_4_ (110 meV Å).^39^ This pronounced Rashba splitting indicates strong spin–orbit interaction combined with structural inversion asymmetry in the material. Previous DFT studies have shown that the Rashba splitting in Janus 2D materials can be effectively tuned by an external electric field^40^ or charge doping,^41^ highlighting their potential for electrically controllable spintronic applications. It is also noted that while broken inversion symmetry induces robust spin-splitting, spin-polarized transport depends exclusively on charge carriers near the Fermi level. Such characteristics make the WBPX_2_ monolayers promising candidates for future spintronic technologies where controllable spin polarization is essential.

### 3.3 Carrier mobility

Carrier mobility is a crucial metric for assessing the electronic and optoelectronic potential of semiconductors. In this work, we evaluate the carrier mobility using first-principles inputs within the AMSET code.^32^ This method enables the explicit treatment of different scattering sources, providing a more realistic representation of carrier transport in 2D systems. It should be also noted that the carrier mobility values obtained using AMSET are approximate theoretical estimates based on semi-classical scattering models and are mainly intended to provide insight into the intrinsic carrier behavior and dominant scattering mechanisms of the studied 2D material. In the present calculations, the carrier mobility was limited by four main scattering mechanisms, including Acoustic Deformation Potential (ADP) scattering associated with lattice vibrations, Ionized Impurity (IMP) scattering, Piezoelectric (PIE) scattering, and polar optical phonon scattering. The dielectric constants required for describing the polar optical phonon contribution were obtained from density functional perturbation theory calculations. The total carrier mobility (*µ*_total_) was then derived according to Matthiessen's rule, where the inverse contributions from each scattering process are summed to obtain the overall transport response:^23^3
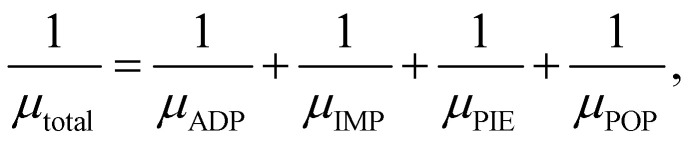
where *µ*_ADP_, *µ*_IMP_, *µ*_PIE_, and *µ*_POP_ refer to the carrier mobilities associated with the ADP, IMP, PIE, and POP scattering mechanisms, respectively.

Previous studies on related MA_2_N_4_-type monolayers have indicated that carrier mobility remains nearly unchanged at low carrier densities but decreases noticeably once the concentration exceeds approximately 1 × 10^19^ cm^−3^,^22^ mainly due to the increased probability of carrier scattering. Following this observation, we selected two representative carrier concentrations, 1 × 10^16^ cm^−3^ and 1 × 10^20^ cm^−3^, corresponding to the low- and high-doping regimes, respectively. Electron and hole mobilities were calculated over a broad temperature range to examine how thermal effects influence scattering strength and transport behavior. This approach enables a consistent assessment of the intrinsic mobility and provides useful insight into the suitability of these Janus monolayers for future electronic and optoelectronic applications.

**Fig. 6:**
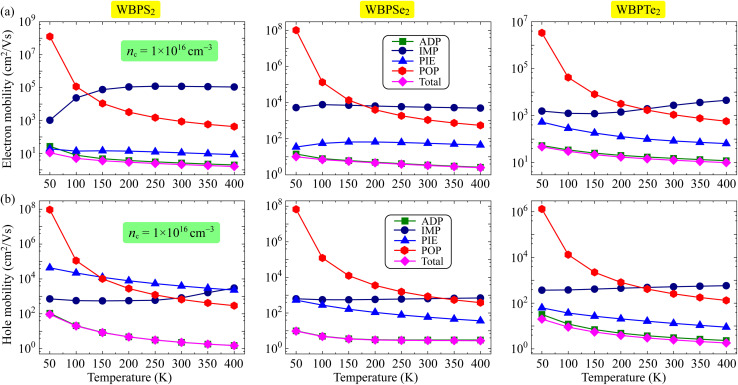
Temperature-dependent electron (a) and hole (b) mobilities of Janus WBPX_2_ monolayers at low carrier concentration of 1 × 10^16^ cm^−3^.

**Fig. 7:**
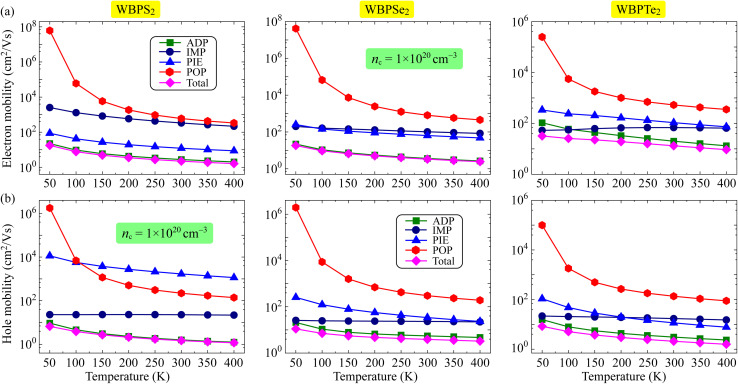
Temperature-dependent electron (a) and hole (b) mobilities of Janus WBPX_2_ monolayers at high carrier concentration of 1 × 10^20^ cm^−3^.

The temperature dependence of electron and hole mobilities in the Janus WBPX_2_ monolayers at different carrier concentrations is presented in Fig. 6 and 7. Calculations were performed over a wide temperature range (50–400 K) to elucidate the underlying scattering mechanisms. The results show that carrier transport is primarily governed by ADP scattering across the considered temperature range, dominating both the low- and high-concentration regimes. In the low carrier concentration regime, PIE scattering also plays a noticeable role in determining the overall mobility, especially at lower temperatures. With increasing temperature, POP scattering becomes increasingly important due to the enhanced population of optical phonons and the resulting stronger electron–phonon coupling. In general, the carrier mobilities limited by ADP, PIE, and POP scattering mechanisms gradually decrease with increasing temperature. By contrast, the mobility associated with IMP scattering exhibits an opposite trend in the low carrier concentration regime, increasing with temperature for both electrons and holes. In the high carrier concentration regime, the relative contributions of different scattering mechanisms change noticeably. Although ADP scattering continues to dominate the transport process, the influence of IMP and PIE scattering becomes increasingly pronounced. In particular, these two mechanisms contribute more significantly to the reduction of carrier mobility than POP scattering under such conditions.

To gain a better understanding of the scattering processes that influence carrier transport at room temperature (*T* = 300 K), we present the individual contributions of the relevant mechanisms in Table 3. The analysis indicates that ADP scattering is the primary factor limiting carrier mobility in the Janus WBPX_2_ monolayers, regardless of whether the carrier concentrations are low or high. Among the investigated systems, the WBPTe_2_ monolayer exhibits the highest electron mobility, achieving values of 12.57 cm^2^ V^−1^ s^−1^ at a carrier concentration of 1 × 10^16^ cm^−3^ and 13.19 cm^2^ V^−1^ s^−1^ at 1 × 10^20^ cm^−3^. Conversely, the WBPSe_2_ monolayer demonstrates the highest hole mobility, with values of 2.86 cm^2^ V^−1^ s^−1^ at low carrier concentration and 3.89 cm^2^ V^−1^ s^−1^ at high carrier concentration. While ADP scattering predominantly governs the overall transport behavior, POP scattering also significantly contributes to the reduction of carrier mobility. This indicates that electron–phonon interactions play a crucial role in determining the transport performance of these Janus systems. Overall, the Janus WBPX_2_ monolayers exhibit relatively modest electron mobility compared with high-mobility 2D semiconductors. Similar transport characteristics have been reported in several 2D materials with limited charge transport efficiency, including HfSiAs_3_H (10.27 cm^2^ V^−1^ s^−1^)^20^ and T-ZrTe_2_ (4.7 cm^2^ V^−1^ s^−1^).^23^ The reduced intrinsic mobility in these Janus systems can be largely attributed to strong electron–phonon coupling. In particular, lattice vibrations together with the structural asymmetry inherent in the Janus configuration enhance carrier scattering, thereby suppressing efficient charge transport.

**Table 3:** Room-temperature carrier mobility *µ* (cm^2^ V^−1^ s^−1^) of Janus WBPX_2_ monolayers at various carrier concentrations *n*_c_ (cm^−3^)

Carrier type	Compound	Carrier concentration	*µ*_ADP_	*µ*_IMP_	*µ*_PIE_	*µ*_POP_	*µ*_total_
Electron	WBPS_2_	*n*_c_ = 1 × 10^16^	2.63	1.19 × 10^5^	10.90	873.00	2.11
	WBPSe_2_		3.53	5570.00	56.00	1100.00	3.31
	WBPTe_2_		15.10	2750.00	83.10	1090.00	12.57
	WBPS_2_	*n*_c_ = 1 × 10^20^	2.78	335.00	12.20	599.00	2.24
	WBPSe_2_		3.64	102.00	63.90	810.00	3.32
	WBPTe_2_		20.00	68.50	107.00	531.00	13.19
Hole	WBPS_2_	*n*_c_ = 1 × 10^16^	2.32	824.00	3910.00	667.00	2.30
	WBPSe_2_		3.03	639.00	58.00	857.00	2.86
	WBPTe_2_		3.09	522.00	13.00	256.00	2.46
	WBPS_2_	*n*_c_ = 1 × 10^20^	1.60	22.80	1690.00	220.00	1.48
	WBPSe_2_		5.51	23.20	34.10	302.00	3.89
	WBPTe_2_		3.07	17.10	11.50	136.00	2.09

## 4 Conclusion

In summary, we have systematically investigated the structural stability, vibrational characteristics, electronic properties, and carrier transport behavior of the Janus WBPX_2_ (X = S, Se, Te) monolayers using first-principles density functional theory calculations. The calculated cohesive energies, phonon spectra, and *ab initio* molecular dynamics simulations confirm the excellent structural stability of all proposed systems. Hybrid HSE06 calculations reveal semiconducting band gaps ranging from 0.96 to 1.26 eV. When spin–orbit coupling is included, pronounced spin splitting emerges in the electronic bands, with splitting energies up to 460 meV and a large Rashba parameter reaching 941.12 meV Å, indicating strong spin–orbit interactions. The sizable Rashba spin splitting and strong SOC in WBPX_2_ monolayers suggest that these materials provide a promising platform for future studies of spin relaxation dynamics, spin lifetimes, and electrically controllable spin transport in low-dimensional spintronic devices. The asymmetric Janus configuration also leads to different work functions on the two opposite surfaces. Notably, the carrier transport properties were analyzed by considering several carrier scattering mechanisms. The results demonstrate that acoustic deformation potential scattering plays the dominant role in limiting both electron and hole mobilities. These findings provide valuable insights into the interplay among structural asymmetry, spin–orbit coupling, and carrier transport in the Janus WBPX_2_ monolayers.

## Conflicts of interest

There are no conflicts of interest to declare.

## Data Availability

The data that support the findings of this study are available from the authors upon reasonable request.
